# α1D Adrenergic Receptor Antagonism Protects Against High Glucose-Induced Mitochondrial Dysfunction and Blood Retinal Barrier Breakdown in ARPE-19 Cells

**DOI:** 10.3390/ijms26030967

**Published:** 2025-01-24

**Authors:** Erika Giuffrida, Chiara Bianca Maria Platania, Francesca Lazzara, Federica Conti, Ludovica Sotera, Filippo Drago, Danushki Herath, Roberto Motterlini, Roberta Foresti, Claudio Bucolo

**Affiliations:** 1 Department of Biomedical and Biotechnological Sciences, School of Medicine, University of Catania, 95125 Catania, Italy; 2 Center for Research in Ocular Pharmacology-CERFO, University of Catania, 95125 Catania, Italy; 3 Faculty of Health, University Paris-Est Créteil, INSERM, IMRB, F-94010 Créteil, France

**Keywords:** diabetic retinopathy, α_1D_ adrenergic receptor, α_2C_ adrenergic receptor, mitochondrial dysfunction, blood retinal barrier breakdown, retinal pigment epithelial cells

## Abstract

Diabetic retinopathy (DR) is a microvascular complication of diabetes mellitus and a leading cause of blindness in the working-age population. Current pharmacological treatments counteract DR’s later stages without targeting the earlier disease phases. Using computational approaches, our group previously identified the α1D and α2C adrenoceptors (α1DR and α2CR) as new putative drug targets for DR. Therefore, the aim of this work was to validate the role of these receptors in an in vitro model of DR, i.e., retinal pigmented epithelial cells (ARPE-19) challenged with high glucose (HG, 50 mM). We examined the effects of selective α1DR and α2CR agonists and antagonists on hyperglycemia-induced mitochondrial dysfunction and blood retinal barrier breakdown. Seahorse XFe was employed to assess the oxygen consumption rate and extracellular acidification rate. The integrity of the ARPE-19 barrier was evaluated through transepithelial electrical resistance measurements and a sodium fluorescein permeability test. α1DR pharmacological modulation through the α1DR antagonist BMY 7378 (0.1–1 µM, 24 h), but not α2CR, significantly attenuated HG-induced mitochondrial dysfunction. BMY 7378 (0.1–1 µM, 48 h) also prevented HG-mediated damage to retinal epithelial integrity. In contrast, the α1DR agonist phenylephrine (1–10 μM, 24 h) further reduced ARPE-19 mitochondrial activity compared to HG, indicating that α1D activation is directly implicated in DR-mediated mitochondrial dysfunction. In conclusion, the current in vitro study validated α1DR as a pharmacological target for DR.

## 1. Introduction

Diabetic retinopathy (DR) is a secondary complication of diabetes mellitus and a leading cause of blindness and visual impairment in the working-age population [1]. The earlier DR phase, called non-proliferative diabetic retinopathy, is clinically distinguished by the occurrence of microaneurysms and intra-retinal hemorrhages, whereas retinal neovascularization is the hallmark of the later stage of the disease, i.e., proliferative diabetic retinopathy (PDR) [2]. Diabetic macular edema (DME) is a complication of PDR caused by accumulation of fluid and lipids in the macula [2]. Intravitreal injections of anti-vascular endothelial growth factor (VEGF) drugs or steroids currently represent the gold-standard treatments for PDR and DME, while no pharmacological treatments for the early stages of the disease have been approved [3,4,5]. In addition, a high percentage of patients respond poorly to anti-VEGF treatments [6]. Therefore, to identify new potential therapeutic strategies for DR, Platania et al. (2018) employed a network pharmacology approach to discover novel DR drug targets [7]. Interestingly, α_1D_ adrenergic (α1DR) and α_2C_ adrenergic (α2CR) receptors were among the identified targets, suggesting their potential involvement in the disease. The α1DR, belonging to the α_1_-adrenoceptor subfamily, is a G_q/11_-coupled receptor. Its activation stimulates phospholipase C (PLC), which in turn leads to the production of the second messengers such as inositol trisphosphate (IP_3_) and diacylglycerol (DAG). Concerning the role of α1DR in the eye, it has been found that this receptor is involved in the regulation of the retinal vascular tone as well as protein secretion in the lacrimal glands of rats [8,9].

The α2CR, a member of the α_2_ adrenergic receptor subfamily, is coupled to G_i/o_ protein. Interestingly, it has been reported that agonists of α_2_ adrenoceptors protect retinal cells in glaucoma, ischemia reperfusion injury, and light-induced photoreceptor models [10]. Although the specific role of α_2C_ adrenoceptors in the eye has not yet been elucidated, these receptors have been found to be expressed in the photoreceptor soma and inner segments of the retina, as well as in the ciliary body and corneal/conjunctival epithelia [11,12].

In DR, the persistent exposure to high levels of glucose leads to functional and structural alterations in the mitochondria of retinal neurons, retinal pigment epithelial cells, and microvascular endothelial cells [13,14,15]. Mitochondrial damage can ultimately lead to the extravasation of cytochrome c and activation of apoptosis-related signaling pathways in these cells, thus contributing to DR progression [14,15]. Hyperglycemia is also known to impair mitochondrial metabolic activity of retinal endothelial and Müller cells as well as of pericytes, as suggested by the decreased oxygen consumption rate (OCR) found in these cells [15]. In this regard, Foresti et al. reported a depression of OCR, which represents mitochondrial respiration, and extracellular acidification rate (ECAR), an index of glycolytic activity, in retinal pigment epithelial (RPE) cells cultured in hyperglycemic conditions [16]. Moreover, it has been demonstrated that hyperglycemia directly affects different components of the RPE barrier, e.g., the active transport of solutes across the membrane [17].

The breakdown of both the inner and outer blood–retinal barrier (BRB) is known to play a key role in DR development [17]. In fact, the BRB is essential in preserving the retinal microenvironment by regulating fluids and molecular movement between the ocular vascular beds and retinal tissues [18]. While the inner BRB (iBRB) is established by tight junctions between retinal capillary endothelial cells, surrounded by pericytes and supported by glial cells, RPE, with its tight junctions, constitutes the outer BRB (oBRB) [18,19].

In the present study, we evaluated for the first time the role of α1DR and α2CR in an in vitro model of DR, i.e., ARPE-19 cells subjected to high glucose (HG), by employing selective ligands of these two receptors as pharmacological tools. We found that α2CR ligands did not exert a significant improvement in the mitochondrial function of ARPE-19 challenged with HG. In contrast, the α1DR-selective antagonist BMY 7378 significantly restored cellular mitochondrial respiration and glycolysis impaired by HG, as demonstrated by the preservation of OCR and ECAR profiles. We also showed that the inhibition of α1DR significantly counteracted the reduction in transepithelial electrical resistance (TEER) and the increase in RPE barrier permeability elicited by HG. These findings indicate that α1DR antagonists could protect retinal pigment epithelial cells from the pathological dysfunctions induced by hyperglycemia, thus suggesting that the α1D adrenergic receptor would be an attractive pharmacological target for the management of DR.

## 2. Results

### 2.1. High Glucose (HG) Affects ARPE-19 Bioenergetic Profile

**Figure 1:**
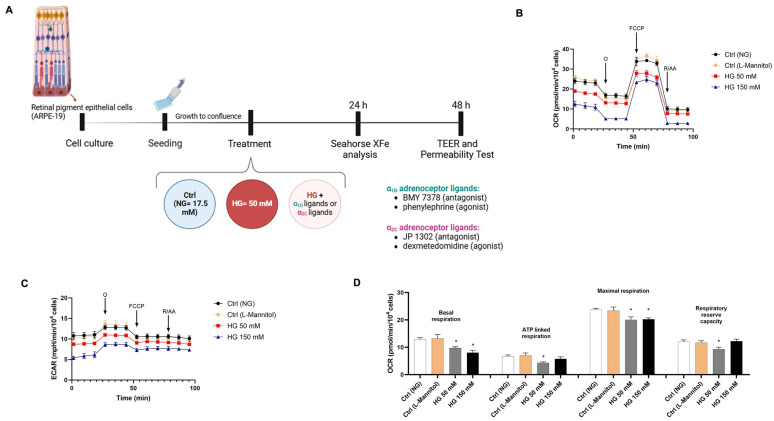
Effect of high glucose (HG) on OCR and ECAR in ARPE-19 cells. (O: oligomycin; FCCP: carbonyl cyanide 4-(trifluoromethoxy) phenylhydrazone; R/AA: rotenone and antimycin A). (**A**) Experimental design for the evaluation of ARPE-19 mitochondria activity and barrier permeability (NG = normal glucose; HG = high glucose). Cells exposed to NG medium were used as control group, while treatment with elevated glucose levels (HG, 50 mM) was employed to mimic DR pathogenesis. ARPE-19 cells were also co-treated with HG and α1DR or α2CR ligands to study the role of the receptors in DR. Seahorse XFe analysis and TEER along with a NaF permeability test were, respectively, carried out after 24 and 48 h of treatment. Exposure of ARPE-19 cells to HG (50 and 150 mM) for 24 h decreased OCR (**B**) and ECAR (**C**) in a concentration-dependent manner. Additional parameters calculated from these curves show that several bioenergetics markers were significantly (*p* < 0.05) affected by exposure to HG compared to control cells (**D**). Values are reported as mean ± SD; *n* = 4. Data were analyzed by one-way and two-way ANOVA and the Tukey post hoc test for multiple comparisons. * *p* < 0.05 vs. control (Ctrl).

The experimental design of the study is summarized in Figure 1A. Different glucose concentrations were initially tested on ARPE-19 to assess cell viability using an MTT assay (Supplementary Materials, Figure S1). We then evaluated the effects of HG (50 and 150 mM) on the mitochondrial activity of ARPE-19. Exposure of ARPE-19 cells to HG for 24 h resulted in impaired mitochondrial function, as shown by a significant (*p* < 0.05) reduction in both OCR (Figure 1B) and ECAR (Figure 1C), indicating that both respiration and glycolytic activity are affected by HG. Moreover, exposure of ARPE-19 cells to HG depressed basal, maximal, and ATP-linked respiration as well as the reserve capacity of mitochondria (Figure 1D). As shown in Figure 1B, cells treated with HG (150 mM) also showed a reduced non-mitochondrial respiration rate in comparison with control and HG (50 mM) treated cells. Furthermore, L-Mannitol did not change the OCR, ECAR, or the bioenergetic parameters of ARPE-19 in comparison with normal glucose (NG) control cells, excluding any influence of osmotic pressure.

### 2.2. Effects of α1D Adrenergic and α2C Adrenergic Receptors Agonists and Antagonists on ARPE-19 Cell Viability Growth in NG

**Figure 2:**
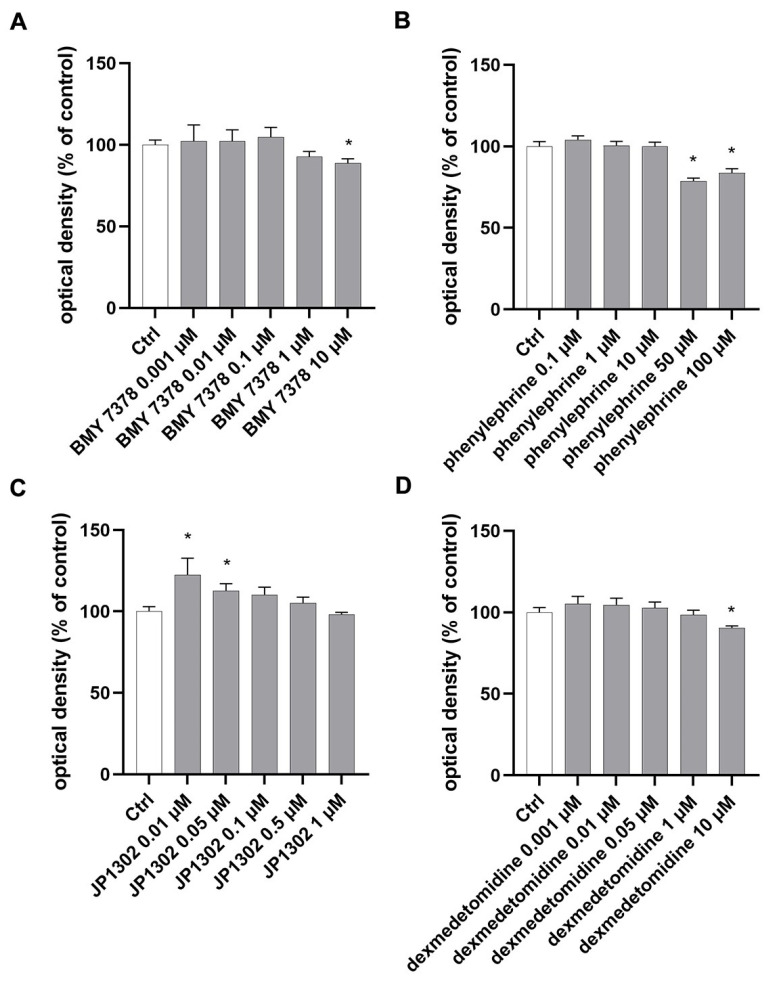
Effect of α1D and α2C receptors agonists/antagonists on ARPE-19 cell viability in NG (MTT assay). Treatment with the α1DR antagonist (BMY 7378 0.001–1 µM) (**A**) and agonist (phenylephrine 0.1–10 µM) (**B**) did not reduce cell viability. Only the higher tested concentrations of BMY 7378 (10 µM) and phenylephrine (50–100 µM) significantly (*p* < 0.05) affected ARPE-19 viability compared to untreated cells (Ctrl). α2CR antagonist, JP 1302 (0.01–1 µM) (**C**), and agonist, dexmedetomidine (0.001–1 µM) (**D**), were tolerated by ARPE-19 cells. Dexmedetomidine at 10 µM led to a significant (*p* < 0.05) reduction in cell viability as compared to control cells (**D**). Values are reported as mean ± SD; *n* = 4. Data were analyzed by one-way ANOVA and the Tukey post hoc test for multiple comparisons. * *p* < 0.05 vs. control.

**Figure 3:**
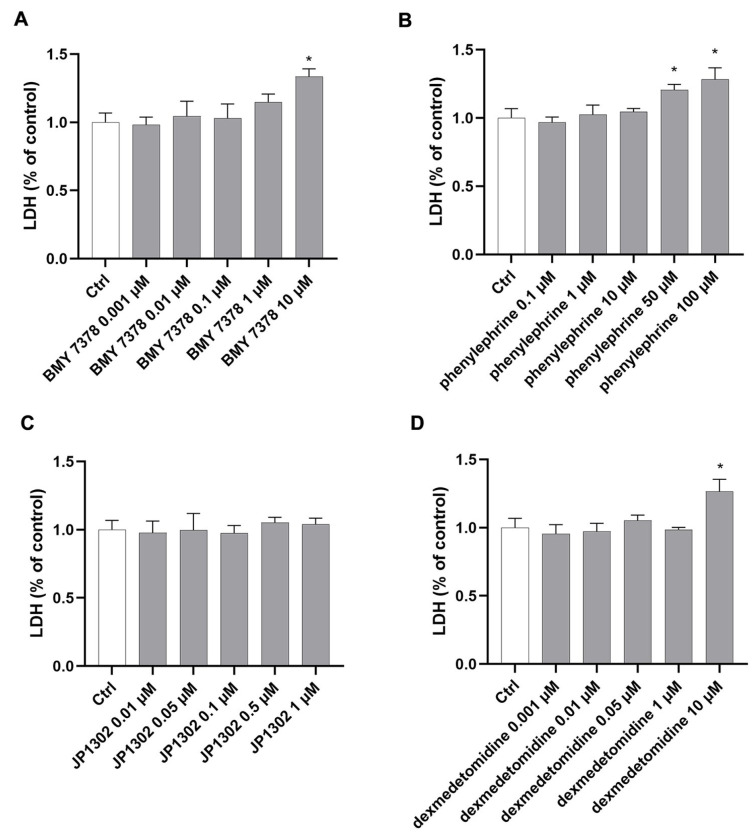
Effect of α1D and α2C receptors agonists/antagonists on ARPE-19 cell viability in NG (LDH Release Assay). Exposure to α1DR antagonist (BMY 7378 0.001–1 µM) (**A**) and agonist (phenylephrine 0.1–10 µM) (**B**) did not increase LDH release compared to control cells. However, treatment with BMY 7378 (10 µM) and phenylephrine (50 and 100 µM) led to a significant (*p* < 0.05) increase in the LDH release as compared to control cells. No significant change in LDH release was detected in ARPE-19 treated with the α2CR antagonist (JP 1302, 0.01–1 µM) (**C**) and agonist (dexmedetomidine, 0.001–1 µM) (**D**). Treatment with the higher tested concentration of dexmedetomidine (10 µM) led to a significant (*p* < 0.05) increase in cell permeability (LDH release) as compared to untreated cells. Values are reported as mean ± SD; *n* = 4. Data were analyzed by one-way ANOVA and the Tukey post hoc test for multiple comparisons. * *p* < 0.05 vs. control.

Preliminary studies were carried out to evaluate the cell viability after 24 h of treatment with α1DR and α2CR ligands. Specifically, an MTT assay was used to assess the cytotoxicity on ARPE-19 cell growth in NG (17.5 mM glucose) of α1DR ligands (BMY 7378 0.001–10 µM and phenylephrine 0.1–100 µM) and α2CR ligands (JP 1302 0.01–1 µM and dexmedetomidine 0.001–10 µM). We found that the α1DR antagonist BMY 7378, at concentrations of 0.001–1 µM, was tolerated by ARPE-19, whereas treatment with BMY 7378 10 µM led to a significant (*p* < 0.05) reduction in cell viability compared to control cells (Figure 2A). Exposure to phenylephrine at 0.1–10 µM did not reduce cell viability. However, phenylephrine at the higher tested concentrations (50 and 100 µM) significantly (*p* < 0.05) decreased ARPE-19 viability as compared to untreated cells (Figure 2B). Concerning α2CR ligands, exposure to the antagonist, JP 1302 (0.01–1 µM), did not affect cell viability compared to control cells. In detail, treatment with JP 1302 at 0.001 and 1 µM led to a significant (*p* < 0.05) increase in cellular viability (Figure 2C). The α2CR agonist (dexmedetomidine 0.001–1 µM) was tolerated by ARPE-19. However, dexmedetomidine at 10 µM led to a significant (*p* < 0.05) reduction in cell viability compared to control cells (Figure 2D). Since the MTT assay primarily measures the metabolic activity of the cells, we evaluated the cytotoxicity of α1DR and α2CR ligands on ARPE-19 cells also by using the lactate dehydrogenase (LDH) release assay (Figure 3). These data confirmed the results generated by MTT (Figure 2).

### 2.3. Effects of α1D Adrenergic and α2C Adrenergic Receptors Agonists and Antagonists on Mitochondrial Respiration and Glycolytic Flux of ARPE-19 Exposed to High Glucose

**Figure 4:**
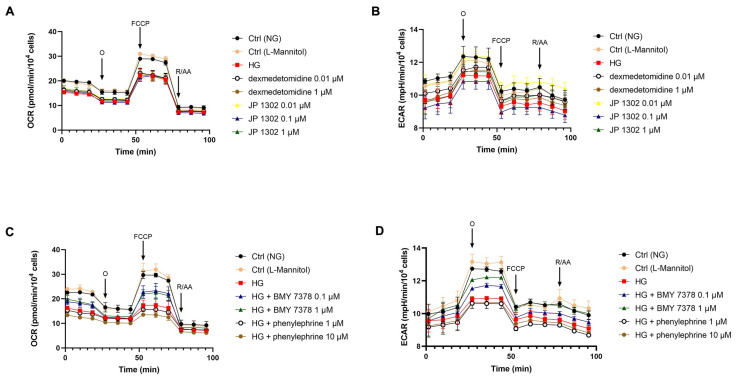
Effect of α_2C_ and α_1D_ receptor agonists/antagonists on OCR and ECAR in ARPE-19 cells exposed to HG. Dexmedetomidine and JP 1302 did not modify the negative effect of HG on mitochondrial respiration, as measured by the oxygen consumption rate (OCR) (**A**). Treatment with α_2C_ receptor agonist (dexmedetomidine 0.01 and 1 µM) did not significantly restore the decrease in ECAR mediated by HG, while exposure to the antagonist JP 1302 (0.01–1 µM) showed a different effect based on the tested concentration (**B**). The α_1D_ receptor antagonist BMY 7378 (0.1 and 1 µM) significantly (*p* < 0.05) restored impairment in cellular OCR induced by HG (**C**) and ECAR (**D**). Phenylephrine exposure (1 and 10 µM) exacerbated the depression of ARPE-19 respiration caused by HG and did not modify ARPE-19 glycolysis compared to HG treatment alone, as indicated by OCR (**C**) and ECAR (**D**), respectively. Values are reported as mean ± SD; *n* = 4. Data were analyzed by two-way ANOVA and the Tukey post hoc test for multiple comparisons.

We then assessed mitochondrial activity after 24 h of exposure of ARPE-19 to different concentrations of the α1DR agonist phenylephrine (1–50 µM) and antagonist BMY 7378 (0.1–10 µM) or the α2CR agonist dexmedetomidine (0.01–10 µM) and antagonist JP 1302 (0.01–1 µM). Mitochondrial activity in ARPE-19 grown in normal glucose concentration (17.5 mM) was not affected by BMY 7378 (0.1–1 µM), phenylephrine (1–10 µM), dexmedetomidine (0.01–1 µM), or JP 1302 (0.01–1 µM). However, due to decreased mitochondrial bioenergetic parameters, 10 µM BMY 7378, 50 µM phenylephrine, and 10 µM dexmedetomidine were excluded in further experiments (Supplementary Material, Figure S2). ARPE-19 cells exposed to HG (50 mM) were then treated with α1DR ligands (phenylephrine 1 and 10 µM or BMY 7378 0.1 and 1 µM) or α2CR ligands (dexmedetomidine 0.01 and 1 µM or JP 1302 0.01, 0.1 and 1 µM). We found that ARPE-19 cells in HG did not modify their OCR profile in the presence of α2C ligands (Figure 4A). Similarly, the dexmedetomidine at 0.01 and 1 µM concentrations did not improve the ECAR profile of ARPE-19 cells challenged with 50 mM glucose for 24 h (Figure 4B). By contrast, 0.01 µM JP 1302 significantly (*p* < 0.05) restored the impaired glycolysis of ARPE-19 induced by HG. However, 0.1 µM of the α2CR antagonist further depressed the HG-mediated decrease in ECAR, and the highest tested concentration (1 µM) showed the same effect of HG (Figure 4B).

Interestingly, treatment with the α1DR antagonist BMY 7378 (0.1 and 1 µM) increased OCR compared to cells exposed to HG alone (Figure 4C), both at basal values and after addition of the uncoupling agent carbonyl cyanide 4-(trifluoromethoxy) phenylhydrazone (FCCP). Exposure to α1DR activator phenylephrine (1 and 10 µM) exacerbated the HG-mediated decrease in OCR, demonstrating a direct role of α1DR activation on ARPE-19 metabolism. Furthermore, these results were confirmed when looking at the ECAR profile as shown in Figure 4D. In fact, treatment with BMY 7378 (0.1 and 1 µM) significantly (*p* < 0.05) improved cellular glycolysis, which was affected by HG. Moreover, phenylephrine (1 and 10 µM) did not significantly affect ARPE-19 glycolytic activity impaired by HG (Figure 4D). Overall, these results suggest that the impairment of bioenergetic profiles by HG in ARPE-19 can be modulated by α1DR ligands. In particular, inhibition of α1DR partially protected against the damage caused by HG. Conversely, the activity of α2CR did not affect the mitochondrial respiration of ARPE-19 exposed to HG. In addition, α2CR had a controversial role in ARPE-19 glycolysis. Only the lower tested concentration of α2CR antagonist significantly (*p* < 0.05) restored the impairment in cellular glycolysis induced by HG. OCR and ECAR data were normalized to the cell number per well and expressed as pmol/min/10^4^ cells and mpH/min/10^4^ cells, respectively.

### 2.4. Effects of α1DR Agonist and Antagonist on Mitochondrial Bioenergetic Parameters in ARPE-19 Exposed to High Glucose

**Figure 5:**
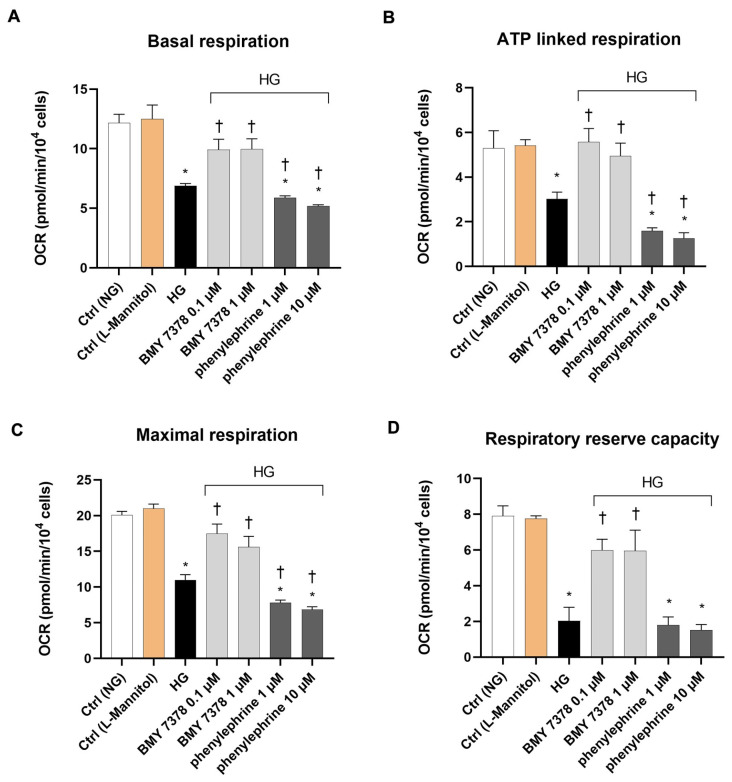
Effect of α1DR agonist and antagonist on bioenergetics parameters in ARPE-19 cells exposed to HG. HG (50 mM) significantly (*p* < 0.05) depressed the bioenergetics parameters quantified from the results of Mito Stress Test. Pharmacological blockade of α1D receptor with BMY 7378 (0.1 and 1 µM) restored HG-impaired basal (**A**), ATP-linked (**B**), and maximal (**C**) respiration. In contrast, exposure to the α1D receptor agonist phenylephrine (1 and 10 µM) significantly (*p* < 0.05) decreased the basal (**A**), ATP-linked (**B**), and maximal (**C**) respiration compared to HG. Treatment with the α1DR antagonist BMY 7378 (0.1 and 10 µM) also significantly (*p* < 0.05) improved the respiratory reserve capacity compared to HG alone (**D**). Phenylephrine (1 and 10 µM), however, did not significantly modify the HG-mediated decrease in respiratory reserve capacity **(D**). Values are reported as mean ± SD; *n* = 4. Data were analyzed by one-way ANOVA and the Tukey post hoc test for multiple comparisons. * *p* < 0.05 vs. control (Ctrl); † *p* < 0.05 vs. HG.

Since α2CR ligands did not modify the effects of HG (50 mM) on respiration and glycolysis in ARPE-19, we calculated the bioenergetics parameters of mitochondrial function only in cells treated with α1DR ligands. ARPE-19 basal respiration impaired by HG was significantly (*p* < 0.05) restored by BMY 7378 (Figure 5A). In contrast, phenylephrine treatment further reduced basal cellular respiration in cells treated with HG. Furthermore, the respiration linked to ATP production, determined after oligomycin injection, was increased to control values by the two concentrations of the α1DR antagonist and decreased by the α1DR agonist (Figure 5B). Moreover, the maximal respiration, which was severely decreased by HG treatment, was significantly (*p* < 0.05) increased by BMY 7378 and reduced by phenylephrine, as shown in Figure 5C. Finally, the spare respiratory capacity, dramatically impaired by HG, was significantly (*p* < 0.05) improved by BMY 7378 and decreased, albeit not significantly, by phenylephrine treatment (Figure 5D).

### 2.5. BMY 7378 Reversed the Effect of Phenylephrine on OCR in ARPE-19 Cells Exposed to High Glucose

**Figure 6:**
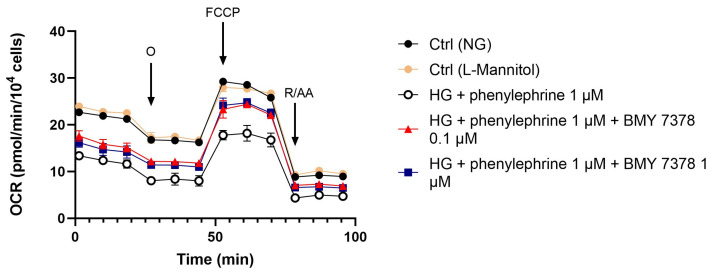
Effect of a combination of α1DR agonist and antagonist on OCR in ARPE-19 cells exposed to HG levels. Treatment with 1 μM phenylephrine (α1DR agonist) significantly (*p* < 0.05) reduced the bioenergetic profile of ARPE-19 cells in 50 mM glucose compared to control cells (Ctrl), as measured by OCR. Co-treatment with the α1DR antagonist BMY 7378 (0.1–1 μM) and phenylephrine (1 μM) for 24 h significantly (*p* < 0.05) restored the phenylephrine-induced decrease in OCR. Values are reported as mean ± SD; *n* = 4. Data were analyzed by two-way ANOVA and the Tukey post hoc test for multiple comparisons.

In a separate set of experiments, we found that treatment with 1 μM phenylephrine decreased the cellular respiration (OCR) compared to control cells. Interestingly, ARPE-19 cells exposed to 50 mM glucose and co-treated with 1 μM phenylephrine (α1DR agonist) and 0.1–1 μM BMY 7378 (α1DR antagonist) exhibited increased OCR compared to cells treated with phenylephrine alone (Figure 6).

### 2.6. BMY 7378 Reversed the Effect of Phenylephrine on Mitochondrial Bioenergetic Parameters in ARPE-19 Exposed to High Glucose

**Figure 7:**
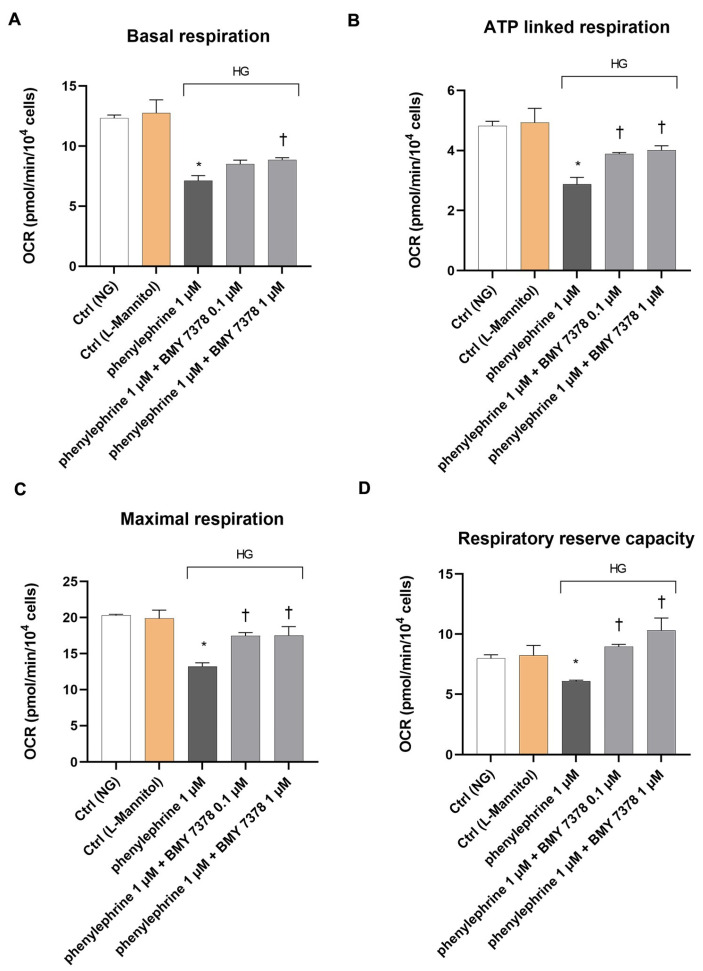
Effect of a combination of α1DR agonist and antagonist on bioenergetics profiles in ARPE-19 cells exposed to HG levels. Co-treatment of ARPE-19 cells exposed to HG (50 mM) with phenylephrine (1 μM) + BMY 7378 (0.1 or 1 µM) for 24 h significantly (*p* < 0.05) reversed the reduction in basal (**A**), ATP-linked (**B**), and maximal (**C**) respiration induced by phenylephrine alone. Similar results were obtained when looking at the respiratory reserve capacity (**D**). Values are reported as mean ± SD; *n* = 4. Data were analyzed by one-way ANOVA and the Tukey post hoc test for multiple comparisons. * *p* < 0.05 vs. control (Ctrl); † *p* < 0.05 vs. phenylephrine 1 μM.

Given that BMY 7378 attenuated the decrease in OCR induced by phenylephrine in ARPE-19 cells challenged with HG, we then analyzed the bioenergetics profiles of these experimental groups. Basal, ATP-linked, and maximal respiration, which were reduced by phenylephrine exposure (1 µM), were significantly (*p* < 0.05) increased by BMY 7378 (0.1–1 μM) (Figure 7A–C). Moreover, the respiratory reserve capacity was significantly (*p* < 0.05) rescued by BMY 7378 in comparison to cells treated with phenylephrine alone (Figure 7D).

### 2.7. Role of α1D Receptor on Outer Blood Retinal Barrier (OBRB) Breakdown Induced by High Glucose

Along with experiments showing the protective effects of the α1DR antagonist on the mitochondrial activity of ARPE-19 exposed to HG, we also tested the effects of α1DR ligands in a model of oBRB breakdown, i.e., the ARPE-19 cell monolayer exposed to HG. We measured the transepithelial electrical resistance (TEER) and the apical-to-basolateral permeability of sodium fluorescein (Na-F) across the epithelial monolayers of ARPE-19 exposed to HG for 48 h and treated with BMY 7378 (0.1 or 1 µM), treated with phenylephrine (1 µM), or co-treated with phenylephrine (1 μM) + BMY 7378 (0.1 or 1 µM). TEER is a quantitative technique which measures the ohmic resistance of a cellular monolayer, while the Na-F permeability test is a measure of permeability across the cellular monolayer. Subsequently to a stressful stimulus, the resistance of a cellular monolayer decreases with the increase in its permeability.

**Figure 8:**
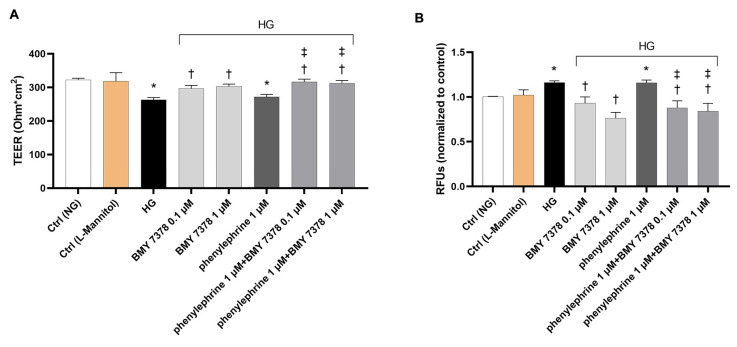
Effect of a combination of α1DR agonist and antagonist on transepithelial electrical resistance (TEER) and apical-to-basolateral permeability in ARPE-19 cells exposed to HG levels. HG levels (50 mM) led to a significant (*p* < 0.05) reduction in the ARPE-19 monolayer integrity compared to control cells, as proven by TEER measurements (**A**). BMY 7378 (0.1–1 µM) significantly (*p* < 0.05) restored the HG-mediated decrease in TEER, while phenylephrine did not modify TEER measurements compared to HG alone (**A**). Phenylephrine (1 μM) + BMY 7378 (0.1 or 1 µM) significantly (*p* < 0.05) increased TEER values compared to HG and to phenylephrine (1 μM) treatments. Measurement of the apical-to-basolateral Na-F permeability revealed a similar pattern to that shown with TEER values (**B**). Representative Na-F permeability measured after 5 min is shown in panel B. Values are reported as mean ± SD; *n* = 4. Data were analyzed by one-way ANOVA and the Tukey post hoc test for multiple comparisons. * *p* < 0.05 vs. control (Ctrl); † *p* < 0.05 vs. HG; ‡ *p* < 0.05 vs. phenylephrine 1 μM.

Accordingly, as shown in Figure 8A, HG significantly (*p* < 0.05) reduced the TEER values of ARPE-19 monolayer compared to control cells. BMY 7378 (0.1 and 1 µM) prevented the decrease in TEER, while phenylephrine (1 µM) did not have any effect on TEER values (Figure 8A) compared to ARPE exposed to HG. In ARPE-19 monolayer, co-treatment with phenylephrine (1 μM) + BMY 7378 (0.1 or 1 µM) also prevented the decrease in TEER values induced by HG. The results regarding TEER values were paralleled by similar findings with the measurement of the apical-to-basolateral permeability of Na-F at all considered time points (5′–15′ and 30′) (Figure 8B).

Therefore, the results of TEER measurements are consistent with the Na-F permeability test and Seahorse data. Also, in the preliminary experiments involving TEER measurements and the Na-F permeability test, we measured the content of proteins for each well and normalized the results. No differences in terms of protein levels were observed in the different groups. Incidentally, we observed the cells before and after the experiments, and no differences in terms of cellular densities were noticed in any of the conditions.

## 3. Discussion

DR is a severe microvascular complication of diabetes mellitus which occurs in 30–40% of diabetic patients [1]. The global prevalence of DR is projected to dramatically increase in the next decades, with an expected growth from 103 million individuals in 2020 to 161 million in 2045 [20]. The lack of drugs able to counteract the earlier stages of DR represents an important unmet medical need. Furthermore, current approved treatments for the proliferative stage of DR, such as anti-VEGF agents, are not only invasive and expensive, but may be ineffective in about 32–66% of unresponsive subjects [21]. In search of novel drug targets to boost the Research and Development (R&D) process for treatment of DR, our group has previously applied a network pharmacology approach [22] to identify innovative targets [7], such as α1D and α2C adrenoreceptors. Therefore, also based on previously encouraging studies demonstrating the involvement of G-proteins coupled receptors (GPCRs) in DR pathogenesis [23,24,25], this work aimed at in vitro validation of α1D and α2C adrenoreceptors as pharmacological targets for the treatment or management of DR. We employed immortalized retinal pigmented epithelial cells (ARPE-19) exposed to HG levels as an in vitro model of DR and, specifically, of oBRB. Different studies have suggested the deleterious effect of HG on RPE cells, including the development of oxidative stress, mitochondrial dysfunction, and apoptosis activation [26,27,28,29,30]. Moreover, it has been shown that the uncoupling of mitochondrial oxidative phosphorylation from ATP synthesis induced by stressful stimuli, including HG, is the main cause of barrier disruption in ARPE-19 cells [31].

We first characterized the effect of HG on mitochondrial respiration and glycolysis in intact ARPE-19 cells by using Seahorse XF technology. We found that HG significantly decreased both OCR and ECAR in ARPE-19, indicating an impairment of electron transport chain function and glycolysis, respectively. Furthermore, basal ATP-linked and maximal respiration, along with the respiratory reserve capacity, were significantly reduced by HG challenge, suggesting a pronounced effect of HG on cellular metabolism. These findings are in line with the results of Foresti et al. (2015), which showed a similar impairment of ARPE-19 cell metabolism after chronic exposure to HG [16]. Moreover, in their experiments, Foresti et al. also found that the mitochondrial bioenergetics parameters were decreased in ARPE-19 cultured in HG (25 mM) compared to cells grown in 5 mM glucose [16]. In accordance with our results, other studies have demonstrated that hyperglycemia leads to mitochondrial dysfunction in RPE cells, e.g., induction of fragmentation, fission, and mitophagy [32,33]. Indeed, we aimed at first to assess the effects of α1D and α2C receptor selective agonists and antagonists on ARPE-19 cells’ mitochondrial function. Concerning α2CR, recent studies have shown that the α2CR agonist dexmedetomidine was able to suppress the overexpression of reactive oxygen species (ROS) and inhibit neural apoptosis using in vitro and in vivo models of cerebral ischemia/reperfusion injury and neuropathic pain [34,35]. Furthermore, dexmedetomidine was also found to restore mitochondrial morphology and function in in vitro and in vivo models of endotoxin-induced acute lung injury and in an in vitro model of hypoxia-activated microglia [36,37]. An agonist of α2 receptors (guanabenz) also decreased superoxide production in retinal cells (661 W cells) subjected to HG (30 mM), leading to a reduction in intracellular cAMP levels [24]. Despite these published findings, it is still unknown whether α2CR contributes to modulate mitochondrial function and cellular bioenergetics of ARPE-19 challenged with HG levels. Because, in our in vitro model of DR, we could not observe significant effects for modulators of the α2CR, we are not able to validate this receptor as a potential pharmacological target for treatment of diabetic retinopathy.

In the case of α1DR, a member of the α_1_ adrenergic receptor subfamily known to mediate vasoconstriction in rat retinal arterioles [8], it has also been reported that these receptors are expressed on the RPE of the rabbit and bovine retina, in which they modulate K^+^ and Cl^−^ transport and electrical currents [10]. Furthermore, the activation of α1 adrenoceptors, as G_q/11_ coupled receptors, stimulates PLC, which leads to the production of the second messenger IP_3_, which is involved in the increase of cytosolic Ca^2+^ concentration. It is worthy to note that the triggering of this signaling pathway has been previously linked to the activation of the NADPH oxidase system [38], a critical pathway in the production of ROS and increased oxidative damage in DR models [39]. Indeed, it has been demonstrated that α_1_ adrenergic receptor antagonists significantly reduce superoxide production and the degeneration of retinal capillaries in in vitro and in vivo models of DR [24,25]. In accordance with these findings, we found that the α1D receptor antagonist BMY 7378 had the ability to restore impaired mitochondrial function in HG-treated ARPE-19, while the α1D receptor agonist phenylephrine decreased cellular OCR under these conditions. In line with our findings, García-Cazarín et al. (2008) [40] demonstrated that the α1 receptor agonist phenylephrine is able to impair the mitochondrial function of human aortic smooth muscle cells through a pathway involving NADPH oxidase, leading to apoptosis of these cells. Moreover, they also found that treatment with the α1DR antagonist BMY 7378 completely prevented the increase in ROS induced by phenylephrine [40].

In our study, the various parameters calculated from the Mito Stress Test reflected the results obtained from the analysis of the bioenergetic profile. Indeed, while BMY 7378 significantly restored the bioenergetic parameters affected by HG treatment, phenylephrine further reduced these parameters compared to HG alone. In line with our findings, it has been found that the α_1_ antagonist prazosin exerted an increase in retinal mitochondrial and cytosolic NAD^+^/NADH ratios in streptozotocin-induced diabetic rats, indicating a beneficial effect on retinal metabolism in diabetic conditions [41]. Furthermore, it has recently been shown that an α1 adrenoceptor antagonist, alfuzosin, improved mitochondrial bioenergetic deficits in the lacrimal glands of diabetic mice induced by norepinephrine [42], thus confirming that the blockade of α1 receptors represents a restorative strategy against mitochondrial dysfunctions. It is important to note that, in our study, ECAR measurements confirmed the trend of the OCR results. In fact, HG-induced reduction in ECAR was restored by BMY 7378 and was not significantly affected by phenylephrine, indicating that the blockade of this receptor also ameliorates glycolytic activities. In this regard, there is evidence for the role of α_1_ antagonists in enhancing glycolysis and increasing cellular ATP levels in another neurodegenerative disease such as Parkinson’s disease [43,44]. Moreover, in our study, the set of experiments testing α1DR antagonist + agonist co-treatment showed that BMY 7378 counteracted mitochondrial dysfunction induced by phenylephrine, as indicated by the increase in OCR and the improvement of mitochondrial bioenergetic parameters. Therefore, these results suggest that BMY 7378 exerts its effect by directly displacing phenylephrine from α1D adrenergic receptors. In line with our findings, other studies have shown that BMY 7378 antagonizes the effects induced by α1DR activation, mediated by phenylephrine, in a competitive manner [45,46].

Our data on mitochondrial functions in ARPE-19 exposed to high glucose levels were coupled with data on the integrity of ARPE-19 monolayer using a model of oBRB, and thus, we assessed the effects of α1D ligands on this parameter. It has already been shown that diabetes increases the permeability across the oBRB and induces a significant loss of integrity of tight junction proteins in RPE tissue [17,47]. Furthermore, hyperglycemia alters RPE barrier function in the earlier stages of DR [48]. In line with these reports, we showed that HG was able to reduce ARPE-19 monolayer TEER and then to increase Na-F permeability compared to control cells. In addition, we found that BMY 7378 restored the retinal epithelial barrier damaged by HG treatment, as proven by instrumental (TEER measurements) and spectroscopic (NaF permeability assays) analyses, while phenylephrine did not show any synergic or additive effects on ARPE-19 monolayer integrity compared to group of cells exposed to HG. We suggest that the positive effects of BMY 7378 on cellular bioenergetics may be linked to a better maintenance of retinal epithelial barrier under HG conditions. Although this is the first study showing α1DR’s involvement in supporting ARPE-19 monolayer integrity, α2 receptor modulation has also been proven to preserve the function of retinal pigmented epithelium barrier and to suppress the upregulation of VEGF in RPE cells challenged with IL-1β [49,50].

The retinal pigmented epithelium is a single layer of hexagonal, polarized, pigmented cells. The retinal pigmented epithelium plays a key role in maintaining normal vision due to its anatomic location between the photoreceptors and the choriocapillaris and the specific biochemical processes that support phototransduction. An essential component in facilitating this function is the tight junction complexes between the cells, which constitute the oBRB. The two most used in vitro models of the retinal pigment epithelium are fetal human RPE (fhRPE) and ARPE-19 cells; however, studies of their barrier properties have produced contradictory results. This could represent a limitation in the use of ARPE-19; however, ARPE-19 cells remain valuable resembling oBRB well accepted by the scientific community. Some labs are making progress on a system in which the interactions of RPE and retinal organoids can be studied, which will be highly beneficial to progress in retinal disease research. We believe that, in the future, innovative systems can be employed to improve retinal barrier models such as retinal organoids or retina-on-a-chip devices established through 3D cellular cocultures or explanted tissues [51,52].

In conclusion, the BMY 7378 α1DR antagonist appears to displace phenylephrine, thus exerting a protective effect against HG-induced mitochondrial dysfunction in ARPE-19 cells. Furthermore, BMY 7378 preserved ARPE-19 monolayer integrity from HG-induced damage. Therefore, our data validated the α1D adrenoreceptor as a potential intriguing pharmacological target for DR management. However, further studies are needed to confirm the involvement of the α1DR receptor for the treatment of DR using knock-out approaches of the *ADR1D* gene in in vivo models of DR.

## 4. Materials and Methods

### 4.1. Cell Culture

Human retinal pigment epithelial cells (ARPE-19) were purchased from ATCC^®^ (Manassas, VA, USA). Cells were cultured at 37 °C, in a humidified atmosphere (5% CO_2_), in DMEM:F12 medium (ATCC number 30–2006, Manassas, VA, USA) with 10% fetal bovine serum (FBS), 100 U/mL penicillin, and 100 μg/mL streptomycin. ARPE-19 cells grown to 70% confluence were used for experimental procedures. All the treatments were carried out in medium with 1% FBS. The DMEM:F12 medium contained glucose at 17.5 mM, and ARPE-19 cells grown in this medium were used as the control group (normal glucose, NG). In addition, ARPE-19 cells were challenged with increased glucose (Sigma-Aldrich, Cat.No. G5400, St Louis, MO, USA) concentrations, i.e., 50 and 150 mM, representing high-glucose (HG) conditions [27,53]. The α_1D_ adrenoceptor ligands BMY 7378 dihydrochloride (Tocris Bioscience, Cat.No. 1006, Bristol, UK) and (R)-(-)-Phenylephrine hydrochloride (Tocris Bioscience, Cat.No. 2838, Bristol, UK) were used at 0.001; 0.01; 0.1; 1.0; 10 µM and 0.1; 1.0; 10; 50; and 100 µM concentrations, respectively. The α_2C_ adrenergic receptors ligands JP 1302 dihydrochloride (Tocris Bioscience, Cat.No. 2666, Bristol, UK) and Dexmedetomidine hydrochloride (Tocris Bioscience, Cat.No. 2749, Bristol, UK) were employed at 0.01; 0.05; 0.1; 0.5; 1 µM and 0.001; 0.01; 0.05; 1.0; 10 µM concentrations, respectively. The tested concentrations were chosen based on the Ki of each ligand for the bound receptor. To rule out potential bias due to an osmotic effect, cells were also exposed to NG medium supplemented with L-mannitol (Sigma Aldrich, St Louis, MO, USA) (17.5 mM glucose + 19.5 mM L-mannitol). ARPE-19 cells exposed to 50 mM glucose were treated with α1DR and α2CR selective ligands for 24 or 48 h.

### 4.2. MTT Assay

The 3-[4,5-dimethylthiazol-2-yl]-2,5-diphenyl tetrasodium bromide (MTT; Chemicon, Temecula, CA, USA) was used to assess cell viability. Cells were seeded in 96-well plates (Costar, Corning, New York, NY, USA) at a density of 2.5 × 10^4^ cells/well to obtain optimal cell density. All the experiments were carried out in DMEM F-12 with 1% of FBS. After reaching confluence (∼70%), ARPE-19 cells were exposed to medium supplemented with 40–150 mM glucose (high glucose, HG), for 24 and 48 h. In another set of experiments, cell cytotoxicity in DMEM F-12 (17.5 mM glucose, NG) was tested for BMY 7378 dihydrochloride (0.001; 0.01; 0.1; 1.0; 10 µM), (R)-(-)-Phenylephrine hydrochloride (0.1; 1.0; 10; 50; 100 µM), JP 1302 dihydrochloride (0.01; 0.05; 0.1; 0.5; 1 µM), or Dexmedetomidine hydrochloride (0.001; 0.01; 0.05; 1.0; 10 µM) after 24 h of exposure. At the end of the treatment, ARPE-19 cells were incubated with MTT (0.5 mg/mL) at 37 °C for 2 h. Then, 100 µL/well of dimethyl sulfoxide (DMSO) (Sigma Aldrich, St Louis, MO, USA) was added, and absorbance was measured at 570 nm in a plate reader (VariosKan, Thermo Fisher Scientific, Waltham, MA, USA). Results were reported as the percentage of control.

### 4.3. LDH Release Assay

LDH cell release was measured by using the CyQUANTTM LDH cytotoxicity assay (C20301, ThermoFisher, Waltham, MA, USA). ARPE-19 cells were seeded at a density of 2.5 × 10^4^ cells/well in Costar 96-well plates. After reaching confluence (∼70%), ARPE-19 cells were treated with BMY 7378 dihydrochloride (0.001–10 µM), (R)-(-)-Phenylephrine hydrochloride (0.1–100 µM), JP 1302 dihydrochloride (0.01–1 µM), or Dexmedetomidine hydrochloride (0.001–10 µM) in NG medium (17.5 mM glucose) for 24 h. According to the manufacturer’s protocol, a lysis solution was then added to positive control wells (non-treated cells) for 45 min. After 24 h, 50 µL of the medium was transferred into a new multiwell, and 50 µL of working solution was added. After 10–15 min at room temperature, 50 μL of stop solution was added. The absorbance values were measured at 490 nm using a plate reader (VarioSkan, Thermo Fisher Scientific, Waltham, MA, USA). LDH release was normalized to control; absorbance values were edited by removing blanks.

### 4.4. Cellular Bioenergetic Analysis Using the Seahorse Bioscience XFe Analyzer

The XFe24 analyzer from Seahorse Bioscience (Billerica, MA, USA) was used to measure the oxygen consumption rate (OCR) for the evaluation of mitochondrial respiration and extracellular acidification rate (ECAR), an index of glycolysis, in real time in ARPE-19 cells. After preliminary experiments, the optimal cell density was obtained by seeding 5 × 10^4^ cells/well on the specific Seahorse XFe24 cell culture 24-well plates (Agilent, Santa Clara, CA, USA). After 24 h of culture, ARPE-19 cells were exposed to HG at concentrations of 50 and 150 mM for 24 h. The α_1D_ antagonist BMY 7378 dihydrochloride (0.1, 1.0 µM) and the α_1D_ agonist (R)-(-)-phenylephrine hydrochloride (1.0, 10 µM) were tested in HG (50 mM) for 24 h. α_1D_ ligands were also used in co-treatment experiments (BMY 7378 dihydrochloride at 0.1 and 1.0 µM and (R)-(-)-phenylephrine hydrochloride at 1.0 µM) in HG (50 mM) for 24 h. ARPE-19 cells were also exposed to JP 1302 dihydrochloride (0.01; 0.1; 1 µM) or dexmedetomidine hydrochloride (0.01; 1 µM) in HG (50 mM) for 24 h. On the day of the assay, the growth medium was replaced with bicarbonate-free low-buffered assay medium according to the manufacturer’s instructions, and a Mito Stress Test was carried out. The Mito Stress assay does not include the addition of 2-DG at the end of the assay, but rotenone and antimycin A; thus, the ECAR results that are shown in the manuscript are those obtained simultaneously with OCR during the Mito Stress assay. Through this test, we determined the following parameters: basal respiration; respiration linked to ATP production after injection of oligomycin (1 µg/mL), an inhibitor of ATP synthesis; maximal respiration after adding the uncoupler FCCP (0.7 µM); and the non-mitochondrial respiration after the simultaneous injection of antimycin A and rotenone (1 µM), which block respiration at the level of complexes III and I, respectively [16]. Base XF medium, carbonyl cyanide 4 (trifluoromethoxy)phenylhydrazone (FCCP), oligomycin, rotenone and antimycin A were obtained from Seahorse Bioscience (Billerica, MA, USA). Finally, the respiratory reserve capacity was calculated as the difference between the maximal and the basal respiration (the difference between OCR after FCCP injection and OCR before oligomycin injection), as previously described [54]. Table 1 shows the equations used to calculate the bioenergetic mitochondrial parameters. OCR and ECAR data were normalized to cell number per well and expressed as pmol/min/10^4^ cells and mpH/min/10^4^ cells, respectively.

### 4.5. Transepithelial Electrical Resistance (TEER) and Permeability Test

The Millicell-Electrical Resistance System (ERS2) (Merck, Millipore, Burlington, MA, USA) was used to measure the transepithelial electrical resistance (TEER) of ARPE-19 cells, as described elsewhere [55,56]. TEER values were expressed as ω × cm^2^ and determined through the following formula: (average resistance of well–average resistance of the blank well) × 0.33 (the area of the membrane). ARPE-19 cells were seeded at 1.8 × 10^5^ cells/well in 12-well plates on cell culture transwell inserts (Falcon^TM^ 12 well 0.4 μm pore size, #353180, Becton Dickinson Labware, Bedford, MA, USA). Cells grown to confluence were treated with BMY 7378 dihydrochloride (0.1, 1.0 µM) and/or (R)-(-)-Phenylephrine hydrochloride (1.0 µM) in HG (50 mM) for 48 h. BRB permeability was evaluated by measuring the apical-to-basolateral movements of sodium fluorescein (Na-F) (Sigma Aldrich, St Louis, MO, USA) across the epithelial cell monolayers. Specifically, a solution of Na-F (10 mg/mL) was added to cell culture inserts transferred into new 12-well plates. Quantification of fluorescence (Na-F: excitation 480 nm, emission 535 nm) was carried out after 5, 15, and 30 min through a Varioskan Flash microplate reader (Thermo Fisher Scientific, Waltham, MA, USA). Values were reported as previously described [57].

### 4.6. Statistical Analysis

Statistical analysis was carried out by using GraphPad Prism 7 (GraphPad software La Jolla, San Diego, CA, USA). Data from all experiments are expressed as mean ± SD (*n* = 4). Assessment of normal distribution of data was carried out with the Shapiro–Wilk test (SPSS, https://www.spss.it/ accessed on 1 March 2024, Crayon Group, Oslo, Norway). One-way and two-way analyses of variance (ANOVA) were performed. Tukey’s post hoc test was used for multiple comparisons. Differences between groups were considered statistically significant for *p*-values  <  0.05.

## Figures and Tables

**Table 1:** Seahorse XFe cell Mito Stress Test parameter equations.

Parameter Value	Equation
Basal respiration	(Last rate measurement before oligomycin injection) − (Non-Mitochondrial Respiration Rate)
ATP-linked respiration	(Last rate measurement before Oligomycin injection) − (Minimum rate measurement after Oligomycin injection)
Maximal respiration	(Maximum rate measurement after FCCP injection) − (Non-Mitochondrial Respiration)
Respiratory reserve capacity	(Maximum rate measurement after FCCP injection) − (Last rate measurement before Oligomycin injection)

## Data Availability

All data generated or analyzed in this study are included in this published article.

## Supplementary Materials

The following supporting information can be downloaded at: https://www.mdpi.com/article/10.3390/ijms26030967/s1.

## Abbreviations

α1DR	α_1D_ adrenergic receptors
α2CR	α_2C_ adrenergic receptors
ARPE-19	Human retinal pigment epithelial cells
BRB	Blood retinal barrier
CTRL	Control
DAG	Diacylglycerol
DME	Diabetic macular edema
DR	Diabetic retinopathy
ECAR	Extracellular acidification rate
FBS	Fetal bovine serum
FCCP	Carbonyl cyanide 4-(trifluoromethoxy) phenylhydrazone
GPCRs	G-proteins coupled receptors
HG	High glucose
iBRB	inner blood–retinal barrier
IP3	Inositol trisphosphate
Na-F	Sodium fluorescein
NG	Normal glucose
oBRB	outer blood–retinal barrier
OCR	Oxygen consumption rate
PDR	Proliferative diabetic retinopathy
PLC	Phospholipase C
R&D	Research and Development
ROS	Reactive oxygen species
RPE	Retinal pigment epithelial
TEER	Transepithelial electrical resistance
VEGF	Vascular endothelial growth factor
