# Supplementary material for: α1D Adrenergic Receptor Antagonism Protects Against High Glucose-Induced Mitochondrial Dysfunction and Blood Retinal Barrier Breakdown in ARPE-19 Cells
Source: Int J Mol Sci. 2025 Jan 24;26(3):967. doi: 10.3390/ijms26030967 (PMC11817144; doi:10.3390/ijms26030967)
Supplement: Supplementary file 1 [file ijms-26-00967-s001.zip › ijms-3402463-supplementary.pdf]

SUPPLEMENTARY MATERIAL

Article

α1D Adrenergic Receptor Antagonism Protects Against High Glucose-Induced Mitochondrial Dysfunction and Blood Retinal Barrier Breakdown in ARPE-19 Cells

Erika Giuffrida[1], Chiara Bianca Maria Platania [1,2], Francesca Lazzara[1], Federica Conti[1], Ludovica Sotera[1], Filippo Drago[1,2], Danushki Herath[3], Roberto Motterlini[3], Roberta Foresti[3,*] and Claudio Bucolo[1,2,*]

1 Department of Biomedical and Biotechnological Sciences, School of Medicine, University of Catania, 95125 Catania, Italy; erika.giuffri@gmail.com (E.G.); chiara.platania@unict.it (C.B.M.P.); francesca.lazzara@unict.it (F.L.); federica.conti@unict.it (F.C.); ludovicasotera@gmail.com (L.S.); fdrago@unict.it (F.D.)

2 Center for Research in Ocular Pharmacology-CERFO, University of Catania, 95125 Catania, Italy

3 Faculty of Health, University Paris-Est Créteil, INSERM, IMRB, F-94010, Créteil, France; danushki.herath@inserm.fr (D.H.); roberto.motterlini@inserm.fr (R.M.)

* Correspondence: roberta.foresti@inserm.fr (R.F.); claudio.bucolo@unict.it (C.B.)

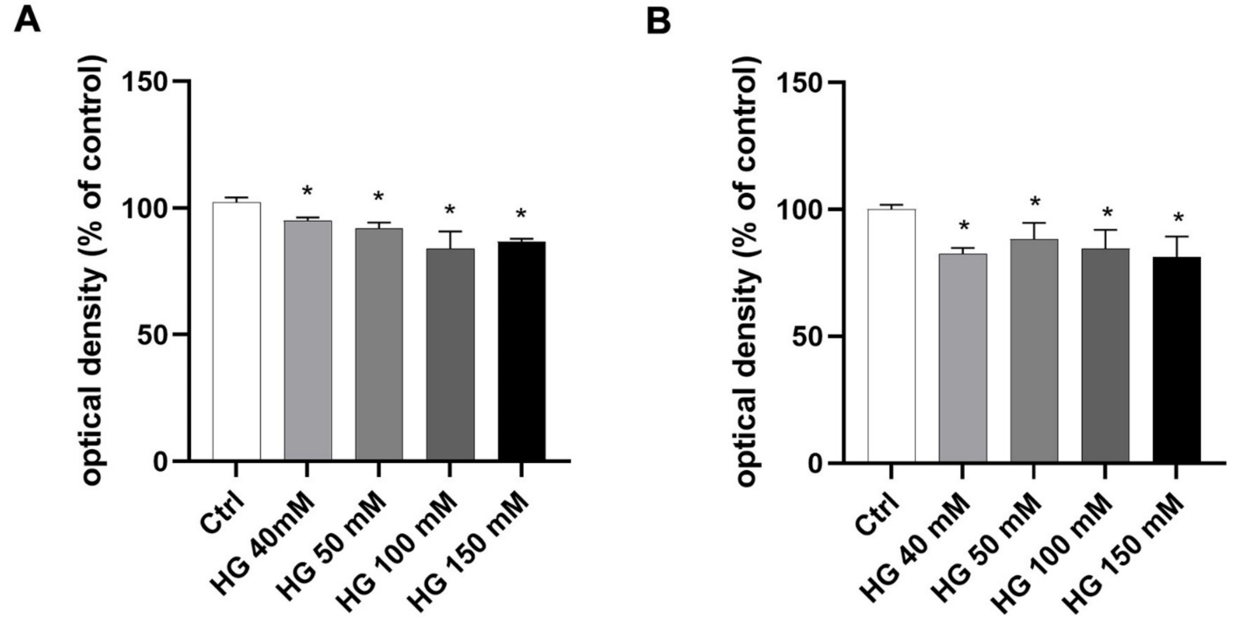

Figure S1: Effect of high glucose (HG) on ARPE-19 Cell Viability after 24 and 48 h of treatment. All the tested HG concentrations (40-150 mM) significantly affected cell viability as compared to untreated (Ctrl) cells after 24h (A) and 48 h of exposure (B). Values are reported as mean ± SD; n = 3. Data were analyzed by one-way ANOVA and the Tukey *post hoc* test for multiple comparisons. *p < 0.05 *vs.* control.

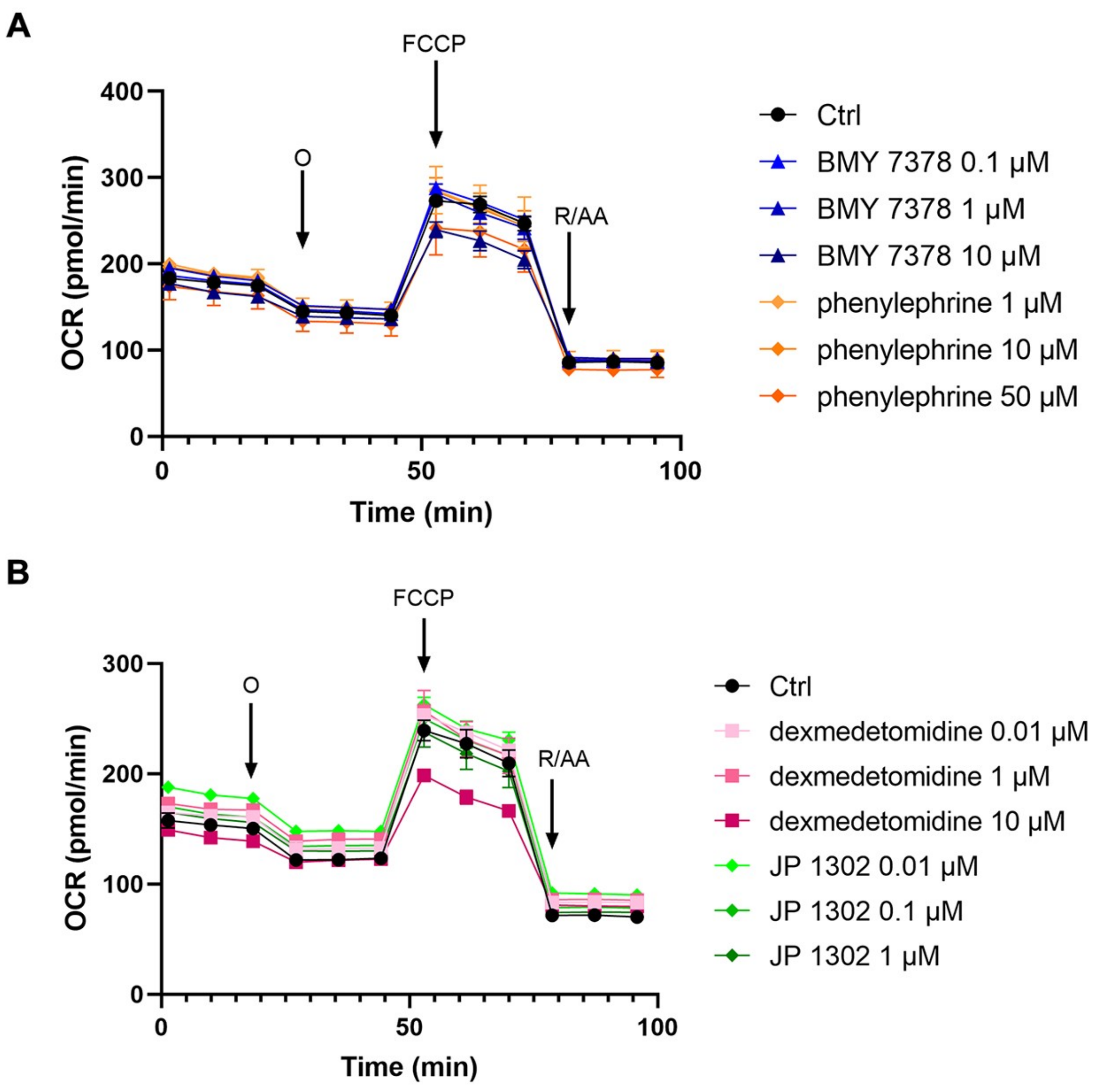

Figure S2: **Effect of α_{2C} and α_{1D} receptor agonists/antagonists on OCR in ARPE-19 cultured in NG (17.5 mM glucose) at 24h of treatment.** BMY 7378 (0.1-1 μM) and phenylephrine (1-10 μM) did not significantly affect the mitochondria activity of ARPE-19 cells growth in NG (17.5 mM), as compared to control cells (A). On the contrary, treatment with BMY 7378 (10 μM) and phenylephrine (50 μM) reduced the OCR compared to Ctrl cells after addition of the uncoupling agent FCCP (A). Exposure to dexmedetomidine (0.01-1 μM) and JP 1302 (0.01-1 μM) did not significantly modify cellular OCR in comparison to control cells (B). By contrast, dexmedetomidine (10 μM) significantly decreased the mitochondrial respiration of ARPE-19 measured after FCCP injection, as compared to control cells (B). Values are reported as mean ± SD; n = 3. Data were analyzed by two-way ANOVA and the Tukey *post hoc* test for multiple comparisons.
